# Supplementary material for: Trends in Hospital Admissions and Death Causes in Patients with Systemic Lupus Erythematosus: Spanish National Registry
Source: J Clin Med. 2021 Dec 8;10(24):5749. doi: 10.3390/jcm10245749 (PMC8707218; doi:10.3390/jcm10245749)
Supplement: Supplementary file 1 [file jcm-10-05749-s001.zip › jcm-1498572-SI.pdf]

Table S1. Admission and death causes by categories.

Group			Global	1997-2000	2001-2005	2006-2010	2011-2015
Cardiovascular disease							
	Coronary disease	Admissions	1911 (1.91%)	213 (1.35%)	433 (1.79%)	566 (2.03%)	699 (2.19%)[T]
		Deaths	88 (3.16%)	13 (3.70%)	21 (3.19%)	25 (3.13%)	29 (2.97%)
	Cerebrovascular disease	Admissions	1791 (1.79%)	217 (1.37%)	371 (1.53%)	520 (1.87%)	683 (2.14%)[T]
		Deaths	175 (6.28%)	18 (5.13%)	41 (6.22%)	51 (6.39%)	65 (6.65%)
	Heart failure	Admissions	1671 (1.67%)	187 (1.18%)	312 (1.29%)	473 (1.7%)	699 (2.19%)[T]
		Deaths	150 (5.38%)	17 (4.84%)	27 (4.1%)	35 (4.39%)	71 (7.26%)
	Arterial TE	Admissions	153 (0.15%)	24 (0.15%)	31 (0.13%)	39 (0.14%)	59 (0.18%)
		Deaths	7 (0.25%)	0 (0%)	1 (0.15%)	4 (0.50%)	2 (0.2%)
	Hypertensive kidney disease	Admissions	412 (0.41%)	38 (0.24%)	59 (0.24%)	128 (0.46%)	187(0.58%)[T]
		Deaths	11 (0.39%)	0 (0%)	1 (0.15%)	1 (0.13%	9 (0.92%)
	Arteriosclerosis	Admissions	404 (0.40%)	23 (0.15%)	82 (0.34%)	135 (0.48%)	164 (0.51%)[T]
		Deaths	16 (0.57%)	0 (0%)	4 (0.61%)	7 (0.88%)	5 (0.51%)
	Peripheral arterial disease	Admissions	1135 (1.14%)	76 (0.48%)	160 (0.66%)	235 (0.84%)	245 (0.77%)[T]
		Deaths	68 (2.44%)	7 (1.99%)	15 (2.28%)	23 (2.88%)	20 (2.04%)
Infection							
	Respiratory	Admissions	4107 (4.11%)	485 (3.07%)	889 (3.67%)	1235 (4.43%)	1498 (4.68%)[T]
		Deaths	195 (7%)	23 (6.55%)	44 (6.68%)	58 (7.27%)	70 (7.16%)
	Urinary	Admissions	1962 (1.96%)	231 (1.46%)	419 (1.73%)	564 (2.02%)	748 (2.34%)[T]
		Deaths	28 (1%)	2 (0.57%)	8 (1.21%)	14 (1.75%)	4 (0.41%)
	Sepsis	Admissions	702 (0.7%)	49 (0.31%)	94 (0.39%)	164 (0.59%)	395 (1.24%)[T]
		Deaths	165 (5.92%)	8 (2.28%)	27 (4.1%)	41 (5.14%)	89 (9.1%)[T]

Skin and soft tissue	Admissions	1100 (1.10%)	79 (0.50%)	149 (0.62%)	174 (0.62%)	217 (0.68%)[T]
	Deaths	26 (0.93%)	2 (0.7%)	1 (0.15%)	2 (0.25%)	5 (0.51%)
Abdominal	Admissions	603 (0.6%)	101(0.64%)	207 (0.86%)	298 (1.07%)	494 (1.54%)[T]
	Deaths	16 (0.57%)	4 (1.14%)	7 (1.06%)	5 (0.62%)	10 (1.02%)
Tuberculosis	Admissions	213 (0.21%)	51 (1.46%)	50 (0.21%)	65 (0.23%)	47 (0.15%)[T]
	Deaths	17 (0.61%)	2 (0.57%)	7 (1.06%)	4 (0.50%)	3 (0.31%)
Neoplasm						
Solid organ	Admissions	2457 (2.46%)	170 (1.08%)	468 (1.93%)	737 (2.64%)	1082 (3.83%)[T]
	Deaths	258 (9.26%)	21 (5.98%)	53 (8.04%)	81 (10.15%)	103 (10.53%)[T]
Hematological	Admissions	453 (0.48%)	75 (0.47%)	86 (0.36%)	137 (0.49%)	185 (0.58%)
	Deaths	58 (2.08%)	4 (1.14%)	10 (1.52%)	18 (2.56%)	26 (2.66%)
Benign	Admissions	851 (0.85%)	92 (0.58%)	171 (0.71%)	245 (0.88%)	343 (1.07%)[T]
	Deaths	0 (0%)	0 (0%)	0 (0%)	0 (0%)	0 (0%)
Unknown origin	Admissions	248 (0.25%)	37 (0.23%)	40 (0.16%)	69 (0.25%)	102 (0.32%)
	Deaths	11 (0.39%)	1 (0.28%)	3 (0.46%)	1 (0.13%)	6 (0.61%)
